# Copland’s Medical Dictionary

**Published:** 1847-06

**Authors:** 


6. *Copland's Medical Dictionary.*—A realization of what is meant by a snail's pace, is to be found in the manner of publishing Copland's Dictionary of Practical Medicine, the fifth volume of which was distributed to the fellows of the Massachusetts Medical Society on the late anniversary meeting. It was wishing a gentleman a long life, when a hope was expressed that he might live till the work was delivered in a state of completion, to the members; and with a weighty sense of the burden of an extreme old age, he might reply that such prospective longevity was undesirable. It is to be feared, from the acknowledged fact that the English language is insensibly undergoing changes, quite as remarkable as it has passed through since the age of Chaucer, that the early part of this learned production will not be as much valued by that remote generation of physicians in whose day the last pages will be distributed, as by their patient, dictionary-loving ancestors.—*Bost. Med. and Surg. Jour.*

